# Demographic and Risk-Factor Differences between Users and Non-Users of Unscheduled Healthcare among Pediatric Outpatients with Persistent Asthma

**DOI:** 10.3390/ijerph17082704

**Published:** 2020-04-15

**Authors:** Pavani Rangachari, Dixie D. Griffin, Santu Ghosh, Kathleen R. May

**Affiliations:** 1 Department of Interdisciplinary Health Sciences, College of Allied Health Sciences, Augusta University, Augusta, GA 30912, USA; 2 Division of Allergy-Immunology and Pediatric Rheumatology, Department of Pediatrics, Medical College of Georgia, Augusta University, Augusta, GA 30912, USA; digriffin@augusta.edu (D.D.G.); kamay@augusta.edu (K.R.M.); 3 Department of Population Health Sciences, Medical College of Georgia, Augusta University, Augusta, GA 30912, USA; sghosh@augusta.edu

**Keywords:** pediatric asthma, self-management effectiveness, healthcare utilization, evidence-based practice guidelines, asthma management, persistent asthma, holistic framework

## Abstract

This study assesses differences between users and non-users of unscheduled healthcare for persistent childhood asthma, with regard to select demographic and risk factors. The objectives are to provide important healthcare utilization information and a foundation for future research on self-management effectiveness (SME), informed by a recently developed “holistic framework” for measuring SME in childhood asthma. An 18-month retrospective chart review was conducted on 59 pediatric outpatients with persistent asthma—mild, moderate, or severe, to obtain data on various demographic and risk factors, and healthcare use for each child. The study examined five types of “unscheduled” healthcare use. Users had non-zero encounters (at least one) in any of the five types; non-users had zero encounters (not even one) in all five types. Differences between users and non-users were assessed using contingency table and logistic regression analysis. There were 25 users and 34 non-users of unscheduled healthcare. Each severity category contained users and non-users. The only statistically significant finding was that the mild persistent category had fewer users than severe persistent (*p* < 0.05). There were no significant differences between users and non-users for any other demographic or risk factor examined. After adjusting for asthma severity, there were no other significant differences between users and non-users of unscheduled healthcare. This is a crucial finding which suggests that something else is driving unscheduled healthcare use in these children, given there were users and non-users in each asthma severity category. These results provide impetus for future research on the role of other aspects of the "holistic framework" in explaining differences in uses of unscheduled healthcare in persistent childhood asthma.

## 1. Introduction

Asthma affects over 43 million Americans and is associated with enormous healthcare expenditures, including an estimated $56 billion/year in direct costs [1]. According to the 2007 National Institutes of Health (NIH) National Heart, Lung, and Blood Institute (NHLBI) evidence-based guidelines for asthma management and recent systematic meta-reviews of the literature, use of unscheduled and costly healthcare encounters, such as emergency visits, hospitalizations, or urgent care, can be prevented through effective self-management of asthma [2,3,4,5,6]. The guidelines further emphasize the importance of creating a provider–patient partnership to enable patients/families to regularly monitor asthma symptoms and take control of their disease, so that treatment can be adjusted as needed. In other words, NHLBI guidelines have highlighted the central role of ‘provider–patient/family communication’ in enabling self-agency or ‘patient/family activation’ to promote effective self-management of asthma. Nevertheless, gaps in adherence to NHLBI guidelines in clinical practice continue to be reported in the literature [1,2,3,4].

Independently, research on asthma management has found that higher levels of patient/family activation (knowledge, skills, and confidence) in asthma management are associated with increased medication adherence and symptom control, even in underserved rural communities [7]. Importantly, a recent systematic review of research on supported self-management of asthma found that interventions targeting the combination of patient, provider, and organizational factors demonstrated the greatest improvement in clinical outcomes, compared to those targeting factors specific to patients or physicians alone [4]. Therefore, both NHLBI guidelines and research on asthma management have underscored the need for increasing provider and organizational (hospital/clinic) engagement in improving asthma self-management effectiveness (SME).

For providers and organizations to be more engaged in improving SME of childhood asthma, they need a comprehensive set of resources for measuring SME, which currently does not exist. The fragmented nature of the existing literature on asthma management and disparate definitions of SME have contributed to this gap in the literature. For example, some studies have defined asthma SME in terms of ‘medication adherence,’ while others have defined it solely in terms of ‘healthcare use’ [7,8]. Similarly, while some studies have examined associations between ‘patient/family activation’ and ‘medication adherence,’ others have focused on examining associations between ‘provider–patient/family communication’ and ‘medication adherence’. As such, these studies reveal limitations in consideration of the concurrent influences of ‘provider–patient/family communication’ and ‘patient activation’ on both ‘medication adherence’ and ‘healthcare use’ [7,8]. Previous studies also do not concurrently adjust for the impact of a host of socioecological variables that have been identified as relevant in influencing asthma SME, including individual, interpersonal, socioeconomic, health system (organizational), community-level, and environmental-level factors [9].

### 1.1. A “Holistic Framework” for Measuring SME in Childhood Asthma

Recognizing these gaps in the literature, researchers have recently worked to develop a “holistic framework” for measuring SME in childhood asthma [10]. The rationale behind this framework is as follows:

- Extrinsic socioecological factors (individual demographic and risk factors, interpersonal factors, socioeconomic factors, health system factors including provider–patient/family communication on asthma management, community-level factors, and environmental-level factors), can impact:
- Intrinsic self-agency factors (patient/family activation in asthma management); to ultimately influence
- SME in childhood asthma. SME in turn is defined by both primary outcomes (unscheduled healthcare use in children with asthma) and intermediate outcomes (asthma medication adherence and symptom control).

A figure summarizing the “holistic framework” for measuring SME in childhood asthma, is available in a previously published open-access article by the authors [10]. The intent of the “holistic framework” is to guide empirical research seeking to shed light on a comprehensive set of factors influencing asthma SME, and interrelationships among these factors, to create a robust evidence base for generating resources for providers to measure asthma SME, and improve SME through effective, targeted interventions.

### 1.2. Study Objective

This retrospective study takes a first step towards assessing SME in childhood asthma, informed by the “holistic framework”, by defining SME in terms of the primary outcome of unscheduled healthcare use. In this study, "users" of unscheduled healthcare serve to represent “low SME”, while "non-users" of unscheduled healthcare within the same asthma severity category serve to represent “high SME.” 

The objective of this study is to examine differences between users and non-users of unscheduled healthcare in persistent childhood asthma, in terms of select extrinsic factors informed by the “holistic framework,” specifically variables obtained through records review, including individual demographic and risk factors. By gleaning a better understanding of the individual demographic and risk factors influencing unscheduled healthcare use in childhood asthma, we propose to provide a foundation for future research on SME of childhood asthma.

## 2. Methods 

The setting for this retrospective study was an allergy–immunology specialty outpatient clinic at a children’s medical center, located within a broader public tertiary academic health system in Augusta, Georgia. The study began following approval from the study site’s Institutional Review Board (IRB). A waiver of informed consent for retrospective chart review was granted by the IRB.

A retrospective chart review was conducted on a purposive sample of 59 unique pediatric outpatients (8–17 years) who visited the clinic for follow-up of persistent asthma between February and September 2019. Children were included in the study if they belonged to any of the following three severity categories for persistent asthma, as defined by NHLBI guidelines: mild, moderate, or severe. All had a physician diagnosis of asthma informed by detailed evaluation to categorize severity; each child had pulmonary function test (PFT) data, with PFTs performed according to American Thoracic Society (ATS) criteria.

Exclusion criteria applied prior to finalizing the sample included children with asthma who were initially seen as new patients within the study period, as within-system retrospective data might not be available, and those defined as having intermittent asthma, to allow focus on those with ostensibly higher healthcare utilization over the study timeframe. Additionally, children with the potential for unrelated respiratory disease, including those with cystic fibrosis, congenital cardiac comorbidities, respiratory disease of prematurity, primary immunodeficiency, and neuromuscular disorders were excluded from the study. The chart review was conducted by two study physicians. 

For the 59 children who met study inclusion criteria, data were collected in the following four categories of data elements informed by the SME “holistic framework” (discussed earlier), through an 18-month retrospective review of individual medical records:

- Individual demographic characteristics, including: age (≤12 years or >12 years); gender (male or female); race (Caucasian, African American, or Hispanic); insurance (Medicaid, private, Tricare/military, or self-pay); NHLBI asthma severity category (mild, moderate, or severe persistent);
- Individual risk factors, including: body-mass index (BMI), defined as normal (<85%), overweight (85–95%), or obese (>95%); prescription for an asthma biologic therapy, i.e., monoclonal antibody (yes or no); prescription for allergen subcutaneous immunotherapy (SCIT, yes or no); number of ‘no-shows’ for scheduled clinic visits;
- Primary outcomes of SME, i.e., healthcare use, which was assessed in terms of: a) ED visits; b) inpatient hospitalization; c) pediatric intensive care unit (PICU) hospitalization; d) unscheduled outpatient clinic visits initiated outside of scheduled visits; e) urgent care visits; and f) scheduled outpatient clinic visits;
- Intermediate outcomes of SME, including asthma symptom control measured by age-appropriate Childhood Asthma Control Test (CACT^TM^)or Asthma Control Test (ACT^TM^), noted as poor if score was ≤19, or good if >19; medication adherence, estimated as "low", "fair", or "high" from medical record documentation of prescription adherence during records review.

The first five types of healthcare use (a–e), constituted “unscheduled” healthcare use. “Users” of unscheduled healthcare were defined as children with non-zero encounters (at least one) in any of the five types. “Non-users” of unscheduled healthcare were defined as those with zero encounters (not even one) in all five types, collectively. Differences between users and non-users were assessed with respect to aforementioned individual demographic and risk factors, using contingency table and logistic regression analysis.

## 3. Results

Table 1 summarizes the frequency distribution of users and non-users of unscheduled healthcare in the full dataset, with regard to individual demographic characteristics, other individual risk factors, and intermediate outcomes of SME (i.e., medication adherence and asthma symptom control). As indicated in Table 1, there were 25 users and 34 non-users of unscheduled healthcare encounters in the full dataset of 59 children. As shown in Table 1, each of the three persistent asthma severity categories contained users and non-users. The mild persistent asthma category had four users and 16 non-users, for a total of 20; the moderate persistent asthma category had 11 users and 14 non-users, for a total of 25; and the severe persistent asthma category had 10 users and four non-users, for a total of 14 children. Although there were fewer users in the mild persistent category, compared to severe persistent, it was noteworthy that all three severity categories had users and non-users, with the moderate persistent category having a fairly equal distribution (44% vs. 56%, respectively). 

Table 2 summarizes results from the contingency table analysis (chi-square or Fisher’s Exact test, as applicable) in both the severity-stratified dataset (mild, moderate, or severe persistent asthma), and the full dataset. Table 3 summarizes the results from logistic regression analysis of the full dataset, including the regression coefficient estimates and *p*-values for the parameters.

As shown in Table 2, the only statistically significant variable in the contingency table analysis was “asthma severity” (*p* = 0.008) in the full dataset. This is corroborated by results of logistic regression (Table 3), which clarify that the number of users in the mild persistent category was significantly lower than in the severe persistent category (*p* = 0.0168). However, Table 3 also clarifies that there were no statistically significant differences in number of users between the moderate persistent and severe persistent categories. 

We then proceeded to assess differences between users and non-users of unscheduled healthcare in childhood asthma, with regard to the other demographic and risk factors. As shown in Table 2 and Table 3, results indicate that there were no statistically significant differences between users and non-users on any other demographic or risk factor examined. To reiterate, these factors included age, gender, race, insurance, BMI, asthma biologic prescription, allergen immunotherapy (SCIT), no-shows for scheduled clinic visits, asthma medication adherence, and asthma symptom control. 

In the contingency table analysis (Table 2), only two variables other than “asthma severity” approached significance at the 5% level: (1) asthma biologic prescription (*p* = 0.074), which serves as surrogate for asthma severity; and (2) no-shows for scheduled clinic visits (*p* = 0.0724). The frequency distribution of no-shows in Table 1 helps discern that the no-show frequency was relatively higher among users than non-users. While 20% of users had zero no-shows, 40% of non-users had zero no-shows (i.e. 80% of users missed at least one scheduled visit, whereas 60% of non-users missed at least one scheduled visit). 

However, it must be noted that “no-shows” for scheduled visits was neither statistically significant (*p* > 0.05) in the full dataset, nor was it statistically significant in any of the severity-stratified datasets, as indicated in Table 2. In the logistic regression analysis, no variable other than asthma severity was statistically significant (as indicated in Table 3). In summary, given that there were users and non-users in the full dataset, and separately in each of the three severity categories for persistent asthma, the results from this study suggest that alternative factors, other than the demographic and risk factors examined in this study, may be driving use of unscheduled healthcare in children with persistent asthma. 

For reference purposes, Table 4 provides a frequency distribution of the five types of unscheduled healthcare encounters in the full dataset. 

## 4. Discussion

The only statistically significant finding from the study was there were fewer users in the mild persistent category, compared to the severe persistent category, which is an intuitive and expected finding. However, asthma severity only partially explained differences between users and non-users of unscheduled healthcare in persistent childhood asthma, *given there were users and non-users in each severity category.* After adjusting for asthma severity, none of the individual demographic and risk factors examined were significant in explaining use of unscheduled healthcare in persistent childhood asthma. This is a crucial finding because it suggests that alternative factors are driving use of unscheduled healthcare in childhood asthma, given there were users and non-users in all three asthma severity categories, with a roughly equal distribution of users and non-users in the moderate persistent category. 

Although existing studies have found significant racial disparities in use of unscheduled healthcare for asthma, these studies have focused on patients who visit the ED and/or are hospitalized for asthma care, which includes a significant number of uninsured patients who do not have access to outpatient specialty care [11,12,13,14]. This study contributes to the literature by examining unscheduled healthcare use among insured patients who receive regular outpatient specialty care for persistent childhood asthma. Our results do not indicate significant racial differences in use of unscheduled healthcare among patients in this category.

There are some notable strengths and limitations of this study, as well as implications of these findings for practice and future research in the area of childhood asthma management.

### 4.1. Strengths and Limitations of Retrospective Study

This study has several strengths, including a specialized dataset of demographic characteristics, risk factors, and healthcare use in persistent childhood asthma from an academic allergy–immunology specialty outpatient clinic located within a public tertiary medical facility. Since the clinic is adjoining a children’s medical center, most of the children receive all their care within the institution, including emergency visits and hospitalizations, thus increasing the likelihood we have captured relevant scheduled and unscheduled health care use. The clinic also serves a more severely-ill and underserved demographic, when compared with the general pediatric population. It is also noteworthy that nearly 70% of the clinic patients are African Americans, who visit from both a metropolitan area and surrounding rural counties of Augusta, Georgia.

The medical record review for the study was performed by specialty physicians in asthma care within a tertiary academic health system, which increases the likelihood that asthma severity categories were properly captured, in accordance with the Expert Panel Report-3 (EPR-3) severity classification. 

Although our sample size of 59 patients is not large, it exceeds the convention (rule of thumb) of n ≥30 for significance testing [15]. Additionally, although the three severity-stratified datasets each had sample sizes below 30, the moderate persistent dataset approximated this number, with a total of 25 children. Importantly, care was taken at the time of significance testing to use Fisher’s Exact tests in place of chi-square tests whenever necessary, to account for small cell frequencies in the contingency table analysis. 

Moving beyond statistical significance, the study sample has high practical significance for the following reasons:

- It provides an 18-month *per patient* window into unscheduled healthcare use among all eligible children who serially visited an outpatient specialty clinic for persistent pediatric asthma follow-up care over eight consecutive months (February–September 2019)
- Since we evaluated children with higher asthma severity, the sample provides a basis for understanding unscheduled healthcare use in a more severely ill population.
- The practical relevance of a sample that is representative of a higher-severity patient base is best explained by the 80/20 Pareto principle on healthcare use, i.e., that 80% of unscheduled (costly) healthcare use can be attributed to the 20% severely-ill populace.

Additionally, the study makes methodological contributions by comprehensively defining five different types of unscheduled healthcare use for childhood asthma, including unscheduled outpatient visits. It also accounts for no-shows for scheduled outpatient visits as a risk factor. Furthermore, by distinguishing between users and non-users of unscheduled healthcare using an unambiguous (all-or-none) definition, this study paves the way for future research directed toward developing holistic risk profiles of users and non-users, which in turn, has potential to generate insights into effective interventions for reducing unscheduled healthcare use in childhood asthma.

Limitations of this study include the general limitations of retrospective reviews, which restrict data collection to elements available in the chart, as opposed to all elements needed to understand potential key drivers of SME and use of unscheduled healthcare encounters in childhood asthma (as informed by the “holistic framework”). Additionally, data on medication adherence was limited to subjective assessment using medical record documentation, as opposed to a more objective measure of medication adherence, using for example, comprehensive pharmacy data on prescription refills, or data from an adherence device. 

In a similar vein, the study lacked data on patient/family adherence with environmental allergen trigger controls, which is another important component of an asthma self-management plan, alongside medication adherence. The study is also limited in relying on medical record documentation for urgent care visits within our health system. If these children presented at an outside facility for an unscheduled visit, and the family did not report it to us at their scheduled visit, we would be both unaware and unable to capture these encounters at a later time. Ideally, chart review data could be combined with statewide databases on healthcare use for Medicaid patients to obtain comprehensive data on urgent care visits for this subpopulation. 

Finally, our patient population may not be representative of the general pediatric population in terms of demographic characteristics and asthma severity. With regard to the latter, our severity distribution is skewed higher, compared to the general pediatric asthma population. However, this may also be viewed as a strength of our study, with data providing unique insights into patterns of unscheduled healthcare use in a more severely-ill pediatric asthma population, which may not be as readily obtained from a general pediatric population.

Despite its limitations, this study is innovative in leveraging a specialized dataset to conduct a study on the impact of several individual demographic and risk factors on unscheduled healthcare use, informed by a “holistic framework” for assessing SME in childhood asthma. In doing so, this study takes a first step towards addressing knowledge gaps associated with the measurement of asthma SME. The results are meaningful and provide an impetus for future research targeted towards understanding predictors of unscheduled healthcare use and SME in childhood asthma.

### 4.2. Implications for Practice and Future Research 

Providers and organizations (hospitals/clinics) are in need of resources and tools for measuring (and predicting) use of unscheduled healthcare (a proxy for low SME) in persistent childhood asthma. Two variables contained in the “holistic framework” may be particularly relevant for future research seeking to gain clarity on predictors of unscheduled healthcare use in childhood asthma. These are: (1) provider–patient/family communication regarding asthma management (factor of extrinsic socioecological influences at the health system/organizational level); and (2) patient/family activation in asthma management (factor of intrinsic self-agency) [16,17,18,19,20,21,22,23]. These variables are particularly important for future research efforts, not only because they have been emphasized by national evidence based (NHLBI) asthma management guidelines, but also because they are directly relevant to increasing provider engagement in promoting asthma SME. 

To elaborate on these ideas, future research could assess patient/family activation in asthma management using a previously validated tool, the Patient Activation Measure (PAM) [17,18]. PAM is known to enable assessment of a broad range of elements reflected in patient activation, including the knowledge, skills, beliefs, and behaviors needed to manage a chronic illness. The abbreviated 22-item PAM scale is designed to reflect a developmental model of activation in four stages. The activated patient/family knows how to manage their condition, maintain functioning, and prevent health declines, and they have the skills and the behavioral repertoire to do so. For example, one scaled statement in PAM that is used to assess activation is “I am confident that I can tell when I need to go get medical care and when I can handle a health problem myself.” If future research serves to unearth that non-users display significantly higher levels of patient/family activation compared to users of unscheduled healthcare encounters, such a finding would be actionable information for providers. Strategic interventions could be designed to increase patient activation. For example, higher patient/family activation might be associated with a significantly lower number of no-shows for scheduled clinic visits, which in turn may be associated with significantly lower use of unscheduled healthcare encounters. The latter would serve to corroborate the near significance of “no-shows for scheduled clinic visit” in this study. Providers, in turn, would have necessary evidence to design interventions targeted at reducing the number of “no-shows” among users of unscheduled healthcare, in an effort to increase patient/family activation. 

Importantly, high quality provider–patient/family communication regarding asthma management (assessed through a separate validated patient-reported measure), may also be found to be positively associated with higher patient/family activation in asthma management. If future research efforts are able to confirm such a pattern, such results have the potential to bolster efforts to improve provider–patient/family communication, with an eye to increasing patient/family activation. 

Findings from this study also point to the importance of examining the interrelationship among other extrinsic socioecological variables not included in this study, including family health literacy (under individual factors), social support (under interpersonal factors), and school support (under community-level factors) for asthma management.

The limitations of this study (discussed above) also provide insight into designing effective future studies seeking to develop comprehensive profiles of users and non-users of unscheduled healthcare in childhood asthma, with an eye to gaining insight into effective interventions for reducing unscheduled healthcare and improving asthma SME. For example, a prospective study design has advantages over retrospective study design in its ability to shed light on causal relationships between extrinsic or intrinsic variables of interest and asthma SME, as well as the interrelationships among these variables. Moreover, future studies could incorporate objective measures of environmental allergen trigger avoidance and medication adherence (e.g., through prescription refills obtained from pharmacies), to overcome limitations of subjective medical record data on these variables. Additionally, partnering with health departments to obtain up-to-date data on urgent care visits for Medicaid patients would serve to bolster the validity of data on this particular component of healthcare use, which may not be otherwise adequately captured. 

In summary, future research efforts guided by the “holistic framework” could help to develop robust profiles of users and non-users of unscheduled healthcare in persistent childhood asthma, i.e., patients with “low SME” vs. “high SME,” respectively. However, in order to be able to develop comprehensive profiles of users and non-users of unscheduled healthcare in childhood asthma, it would be necessary to supplement quantitative analysis with qualitative research, e.g., interviews and focus groups with select users and non-users of unscheduled healthcare (and their asthma providers), to gain a comprehensive understanding of the barriers and facilitators to effective self- management of asthma encountered by users and non-users in the context of daily living. Overall, future study in this area could help to create a comprehensive evidence base for providers and organizations (hospitals/clinics) to measure SME and improve SME in childhood asthma, through effective, targeted interventions.

## 5. Conclusions

This study found no statistically significant differences between users and non-users of unscheduled healthcare encounters in persistent childhood asthma with respect to any of the demographic or risk factors examined in this study, with the exception of asthma severity, i.e., fewer users in the mild persistent category, compared to severe persistent asthma. The latter is an intuitive finding, which reinforces the expectation that there would be significantly more users of unscheduled healthcare in severe persistent vs. mild persistent asthma. However, given that users and non-users were found not only in the full dataset, but also in each of the three asthma severity categories with a roughly equal distribution of users and non-users in the moderate persistent severity category, the question became: were any differences noted between users and non-users for any of the other demographic or risk factors examined? Somewhat surprisingly, there were no statistically significant differences between users and non-users of unscheduled asthma healthcare encounters on any other demographic and risk factors examined in this study. These factors included age, gender, race, insurance type, BMI, asthma biologic prescription, allergen SCIT, no-shows for scheduled clinic visits, asthma medication adherence, and asthma symptom control.

Results from this study provide impetus for future research on the role of other aspects of the SME “holistic framework” not included in this study, to explain differential use of unscheduled healthcare in persistent childhood asthma (and by proxy, differences in SME of childhood asthma). This would include extrinsic health system influences like provider–patient/family communication of asthma management, and intrinsic self-agency factors such as patient/family activation for asthma management, in addition to a host of other socioecological factors having the potential to influence asthma SME at the individual, interpersonal, health system, community, and environmental levels. Future research seeking to leverage the “holistic framework” to gain clarity regarding the key drivers of unscheduled healthcare use in persistent childhood asthma has the potential to produce an evidence base that could generate resources for providers to measure and improve asthma SME. Important information gained could ultimately bolster provider and organizational (system) engagement in improving self-management effectiveness in persistent childhood asthma.

## Figures and Tables

**Table 1:** Frequency distribution of users and non-users by demographic and risk factors.

	Full Dataset			
	"Users" (n = 25)	"Non-Users" (n = 34)	Total (n = 59)	% of Total
***Individual Patient Demographics***				
Age: ≤12 years	11	21	32	54%
Age: >12 years	14	13	27	46%
Gender: Male	17	24	41	69%
Gender: Female	8	10	18	31%
Race: Caucasian	4	7	11	19%
Race: African American	17	24	41	69%
Race: Hispanic	4	3	7	12%
Insurance: Medicaid	17	23	40	68%
Insurance: Private	7	9	16	27%
Insurance: Tricare	1	0	1	2%
Insurance: Self-Pay	0	2	2	3%
Asthma Severity: MILD Persistent	4	16	20	34%
Asthma Severity: MODERATE Persistent	11	14	25	42%
Asthma Severity: SEVERE Persistent	10	4	14	24%
***Individual Patient Risk Factors***				
BMI: Normal	7	16	23	39%
BMI: Overweight	8	5	13	22%
BMI: Obese	10	13	23	39%
Asthma Biologic-Therapy: Yes	5	1	6	10%
Asthma Biologic-Therapy: No	20	33	53	90%
Allergen SCIT: Yes	4	9	13	22%
Allergen SCIT: No	21	25	46	78%
No-Shows for Scheduled Clinic: ZERO	5	14	19	32%
No-Shows for Scheduled Clinic: ONE	5	7	12	20%
No-Shows for Scheduled Clinic: TWO	2	5	7	12%
No-Shows for Scheduled Clinic: THREE	1	4	5	8%
No-Shows for Scheduled Clinic: FOUR	5	4	9	15%
No-Shows for Scheduled Clinic: FIVE	3	0	3	5%
No-Shows for Scheduled Clinic: SIX	1	0	1	2%
No-Shows for Scheduled Clinic: SEVEN	1	0	1	2%
No-Shows for Scheduled Clinic: EIGHT	2	0	2	3%
***Intermediate Outcomes of SME***				
Medication Adherence: Low	7	10	17	29%
Medication Adherence: Fair	7	5	12	20%
Medication Adherence: High	11	19	30	51%
Asthma Symptom Control: Poor Control	13	16	29	49%
Asthma Symptom Control: Good Control	12	18	30	51%

**Table 2:** Differences between users and non-users of unscheduled healthcare in childhood asthma, by individual demographic and risk factors: results from contingency table analysis.

	Full Dataset (n = 59)	Mild Persistent (n = 20)	Moderate Persistent (n = 25)	Severe Persistent (n = 14)
	*p*-Value	*p*-Value	*p*-Value	*p*-Value
***Individual Patient Demographics***				
Age	0.148	0.549 ¥	0.413 ¥	0.176 ¥
Gender	0.831	1.000 ¥	0.389 ¥	1.000 ¥
Race	1.000 ¥	0.514	1.000 ¥	0.203 ¥
Insurance	0.538 ¥	0.727 ¥	1.000 ¥	1.000 ¥
Asthma Severity	0.008 *	N/A	N/A	N/A
***Individual Patient Risk Factors***				
Body-Mass Index (BMI)	0.153	0.418 ¥	0.387 ¥	0.097 ¥
Asthma Biologic Prescription	0.074 ¥	0.793 ¥	0.440 ¥	1.000 ¥
Allergen SCIT Prescription	0.334 ¥	1.000 ¥	1.000 ¥	0.874 ¥
No-Shows for Scheduled Clinic	0.072 ¥	0.896 ¥	0.822 ¥	0.844 ¥
***Intermediate Outcomes of SME***				
Medication Adherence	0.434	0.636 ¥	0.601 ¥	0.748 ¥
Asthma Symptom Control	0.512	1.000 ¥	0.684 ¥	0.222 ¥

* Statistically significant at 5% level. ¥ Fishers Exact Test Used in place of Chi-Square test. N/A: Not Applicable.

**Table 3:** Summary of results from logistic regression analysis.

Logistic Regression Dependent Variable: "User" = 1, "Non-user" = 0			
Parameter	Category ¥	Coefficient Estimate	*p*-Value
Asthma Severity	Mild	−1.4871	0.0168 *
	Moderate	0.0039	0.9936
Age	Age ≤ 12	0.2364	0.5612
Gender	Female	−3.2491	0.9736
Race	African American	3.0411	0.9753
	Hispanic	2.4757	0.9857
BMI	Normal	−0.4561	0.3888
	Overweight	0.6793	0.2301
Allergen SCIT	No	−0.3441	0.5341
Medication Adherence	Fair	0.2361	0.7078
	High	−0.4458	0.3736
Asthma Symptom Control	Good Control	0.2308	0.5354

¥ The excluded category is the baseline category for comparison. * Statistically significant at 5% level.

**Table 4:** Frequency distribution of unscheduled healthcare use.

Unscheduled Healthcare Use	Frequency	%
***ED Visit***		
ED Visit: ZERO	39	66%
ED Visit: ONE	13	22%
ED Visit: TWO	2	3%
ED Visit: THREE	1	2%
ED Visit: FOUR	2	3%
ED Visit: SIX	1	2%
ED Visit: EIGHT	1	2%
***Inpatient Admissions***		
Inpatient Admission: ZERO	51	86%
Inpatient Admission: ONE	8	14%
***PICU Stay***		
PICU Stay: ZERO	56	95%
PICU Stay: ONE	1	2%
PICU Stay: THREE	1	2%
PICU Stay: FOUR	1	2%
***Unscheduled Outpatient Clinic Visit***		
Unscheduled Clinic Visit: ZERO	55	93%
Unscheduled Clinic Visit: ONE	4	7%
***Urgent Care Visit***		
Urgent Care Visit: ZERO	47	80%
Urgent Care Visit: ONE	8	14%
Urgent Care Visit: TWO	1	2%
Urgent Care Visit: THREE	1	2%
Urgent Care Visit: FOUR	1	2%
Urgent Care Visit: FIVE	1	2%
